# Synthesis of Arbitrary Interference Patterns Using a Single Galvanometric Mirror and Its Application to Structured Illumination Microscopy

**DOI:** 10.1021/acsphotonics.5c00516

**Published:** 2025-06-19

**Authors:** Ke Guo, Abderrahim Boualam, James D. Manton, Christopher J. Rowlands

**Affiliations:** † Department of Bioengineering, 4615 Imperial College London, Exhibition Road, London SW7 2AZ, U.K.; ‡ 47694 MRC Laboratory of Molecular Biology, Francis Crick Avenue, Cambridge CB2 0QH, U.K.

**Keywords:** structured illumination microscopy, interference, fringe projection, super-resolution, galvanometer, microscopy, high-throughput imaging

## Abstract

Structured illumination microscopy (SIM) overcomes the diffraction limit of optical microscopy by projecting finely spaced interference fringes with different orientations and phases onto the sample and imaging the result. A major challenge of SIM is to generate the different illumination patterns with a high contrast and switching speed, which commonly requires expensive devices and the sacrifice of illumination power efficiency. We present a new way of generating interference patterns for 2D and 3D SIM achromatically, with high speed and high power efficiency, using only one moving part. The interference patterns are created by a common-path interferometer, with the orientation, polarization, and phase of interference patterns controlled by a single galvanometric mirror. We characterize the contrast and switching speed of the interference patterns and demonstrate their utility by performing high-speed (980 raw frames per second) 2D SIM imaging on fluorescent nanoparticles and 3D SIM on fixed iFluor 488 phalloidin-stained U-2 OS cells.

## Graphical abstract

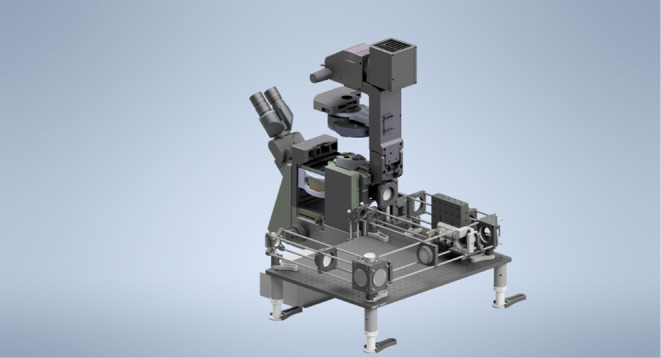

## Introduction

The resolution of an optical imaging system is constrained by the diffraction limit, which dictates how tightly waves may be focused. The diffraction limit is proportional to the product of the wavelength divided by the numerical aperture (itself the product of the immersion medium’s refractive index and the sine of the marginal ray angle), so for most of the 20th century it was commonly believed—because the largest value the sine function can take is one—that for a given wavelength, the only way to increase the imaging resolution was to increase the refractive index. This all changed with the advent of three super-resolution technologies: single molecule localization microscopy,^1,2^ stimulated emission depletion microscopy,^3^ and finally structured illumination microscopy,^4^ which is the focus of this work.

SIM works by projecting a series of finely spaced interference fringes onto the sample, traditionally at three different orientations and with three^4^ or five^5^ different phases per orientation. Suitable weighted combinations of the resulting fluorescence images enable the high spatial frequency content beyond the diffraction limit to be isolated, and recombined to yield the super-resolved image. While it can only increase the resolution up to a factor of 2, compared to other super-resolution methods, SIM has the advantage of requiring a much lower photodose per super-resolved image, it exhibits much higher pixel throughput, and retains compatibility with almost all fluorescent probes, as well as various other imaging techniques such as total internal reflection fluorescence (TIRF)^6^ and light sheet fluorescence microscopy.^7^

There are various ways to create the interference patterns used in SIM. One common approach is to directly project an interference pattern generated by a diffraction grating,^4^ spatial light modulator (SLM)^6^ or digital multimirror device (DMD).^8,9^ These can yield stable interference fringes, but are typically power-inefficient due to higher order diffraction losses;^8,10^ additionally, a different pattern is required for every excitation wavelength, achieving the optimal polarization for maximum fringe contrast is complicated, and the size and pixelation of SLMs and DMDs limits the field of view and resolution. A more photon-efficient (and achromatic) approach is to project a small number of point-sources through a lens; by the Fourier transform properties of a lens, these point-sources become plane waves, which can interfere at the sample to form the desired pattern. The orientation, polarization, amplitude, contrast and phase of the interference pattern can be set by controlling the position, polarization, amplitude, amplitude difference and phase of each corresponding point-source. This implies the need for a separate actuator for each parameter; previous systems using this approach employed a suite of galvanometric (”galvo”) mirrors,^11^ individual piezo-shifted gratings^12^ or individually addressed optical fibers.^13,14^ Unfortunately, using multiple galvos increases cost and introduces instability and synchronization issues. A recent single-galvo solution was achieved using Michelson interferometers.^15^ However, it is limited to 2-beam interference and therefore cannot be used for 3D SIM. As for fiber-based SIM systems, optical fibers have a low damage threshold (limiting light throughput), and fiber components operating at visible wavelengths are significantly more rare and more expensive than their telecoms-wavelength equivalents. What is needed is a simple, high-throughput, achromatic, scalable method that uses a limited number of fast moving parts to perform 2D and 3D SIM.

In this work, we present a new method for generating interference patterns for SIM that we call the Synthetic Wide-field Interfering Foci Technique, or SWIFT. It uses just one galvo mirror to simultaneously control the orientation, polarization and phase of the interference pattern from multiple beams, and thus is capable of high switching speeds which were previously only the preserve of expensive display technologies. Furthermore, it is achromatic, allows for high light throughput, does not suffer from higher order diffraction, and does not suffer from limited field-of-view or resolution caused by finite numbers of projected pixels (although limited numbers of camera pixels will, of course, still be a constraint).

The design of SWIFT is illustrated in Figure 1. In brief, a galvo steers a set of mutually coherent beams to illuminate a column of mirrors. Each mirror steers its beam to an arbitrary lens in a lens array (e.g., the two mirror-lens pairs highlighted in red and green in Figure 1), thus forming a series of foci at the back aperture of a large-aperture lens. The rays from each focus are collimated by this large-aperture lens, forming a desired pattern as they interfere. The angle of each mirror controls which lens array element is illuminated and therefore determines the resulting interference pattern. To change the interference pattern dynamically, another column of mirrors is placed adjacent to the first, each of which steers the incident light to a different lens; turning the galvo to illuminate the new column of mirrors ultimately creates a different interference pattern. More subtly, because the galvo is positioned conjugate to the back aperture of the lens, a small rotation of the galvo would result in a small phase shift of each beam. Such phase shifts are proportional to the horizontal tilts of the beams, which are slightly different from each other. Thus, the phase of the resulting interference pattern can also be changed by the galvo. Finally, by placing a quarter-wave plate and an attenuator over each mirror, the amplitude and polarization of every point source can be individually controlled, thus enabling the control of the polarization and contrast of the interference pattern.

**Figure 1:**
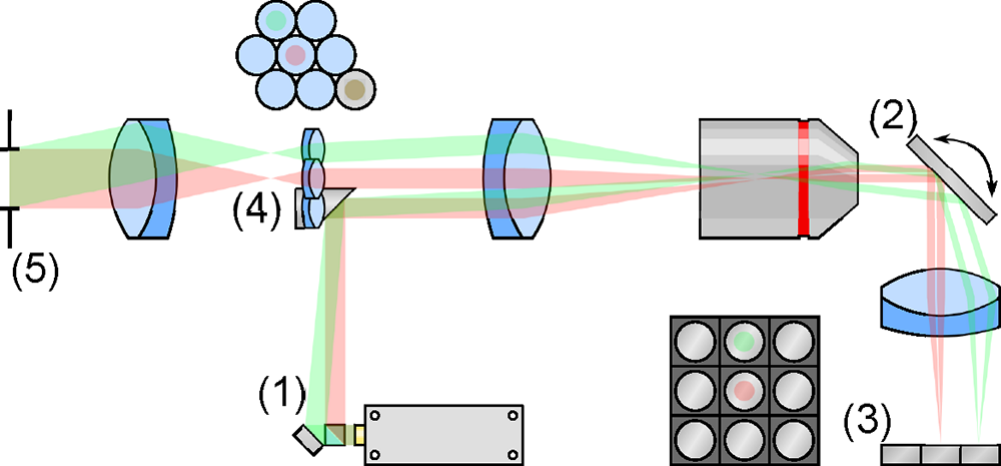
SWIFT design. Light from a laser at (1) is split into multiple beams (only two are illustrated for clarity, in red and green), which are reflected onto the main optical path via a mirror. The beams are imaged and demagnified onto a galvo mirror at (2). The galvo steers each beam through an achromatic lens onto an array of miniature mirrors mounted on small kinematic platforms at (3). Each of the platforms can be controlled independently, to reflect the beam to an arbitrary location on the galvo. The galvo is in a conjugate plane to a miniaturized lens array at (4); the purpose of the miniature mirrors is therefore to steer the retroreflected beams to any of the miniature lenses in the array. The focal plane of this lens array lies at the back focal plane of a achromatic lens, which forms the desired interference pattern at (5). Controlling the galvo at (2) allows the user to illuminate a different column of miniature mirrors, which in turn allow a different set of lenses to be illuminated. In this way, the user can pick between a collection of preset arbitrary interference patterns at will. By taking very small steps with the galvo, the relative phase of the interfering beams can also be controlled.

To demonstrate the capabilities of SWIFT, we first characterize the modulation contrast of the interference patterns, as well as their settling times, before demonstrating both 2D TIRF-SIM imaging of fluorescent nanoparticles at 89 fps (980 fps raw frame rate), and 3D-SIM imaging of iFluor 488 phalloidin-stained U-2 OS cells.

## Materials and Methods

### SWIFT Instrument

A 500 mW 473 nm DPSS laser (LaserQuantum Gem 473) was projected through a variable beam expander before entering a three-way beamsplitter array composed of half wave plates (Thorlabs FBR-AH1) and polarizing cube beamsplitters (Thorlabs PBS051 mounted on FBTC mounts). The reflected s-polarized beams are combined into a set of three closely spaced parallel rays using pick-off mirrors (Thorlabs FBT-P01) before passing through a small Glan Laser prism (Thorlabs GTH5M-A) to improve the polarization purity. An illustration of this beamsplitter array can be seen in Figure 2.

**Figure 2:**
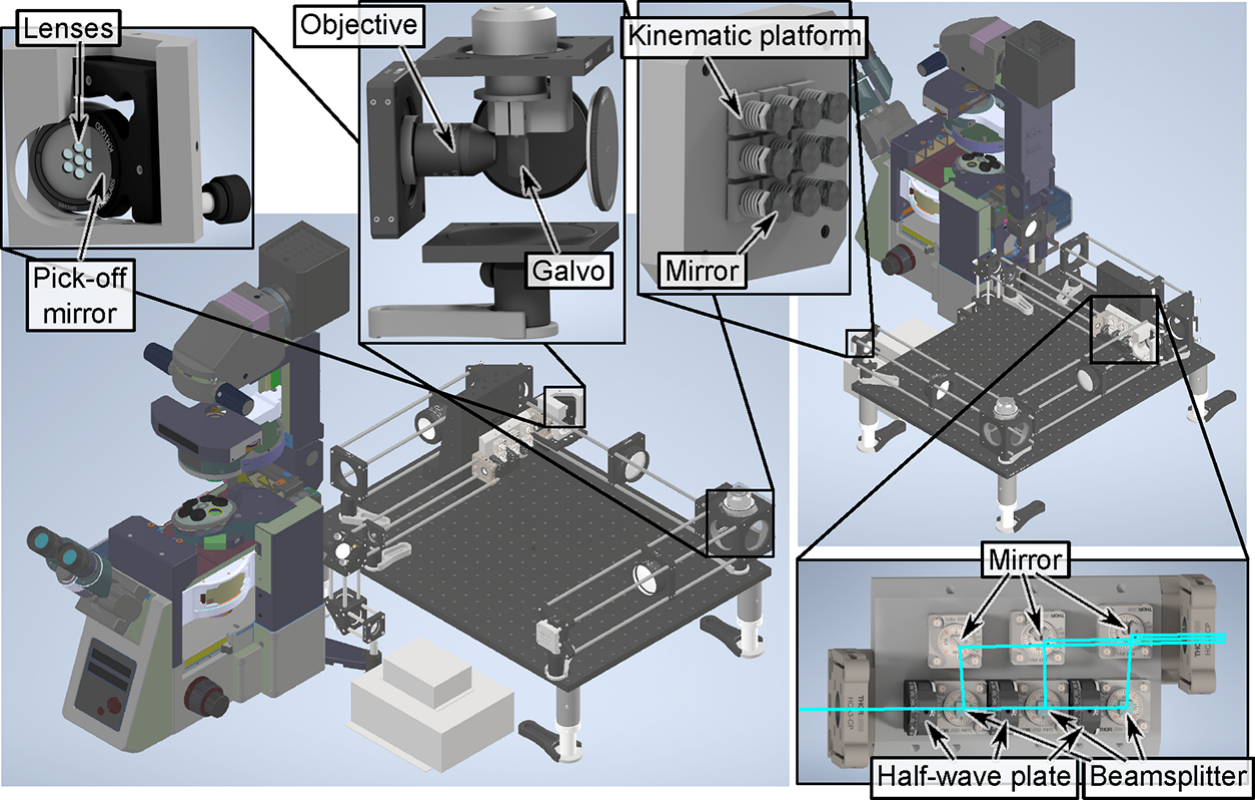
Rendered models of the SIM system (front and back) with important components highlighted. Insets clockwise from top left: *Lens array*, showing the individual lenses held in a custom support, with pick-off mirror positioned adjacent. *Galvo mirror*, with objective focused onto the surface. *Kinematic mirror array* consisting of a 3 × 3 grid of mirrors each mounted on custom 3-axis kinematic mounts. *Beamsplitter array*; the cyan beam entering on the bottom left passes through three sets of half-wave plates and polarizers, which control the relative power of the reflected beams. These beams (each nominally s-polarized) pass through a Glan-Laser prism (not visible) to improve the polarization purity.

The three beams are reflected from a pick-off mirror (Thorlabs BBD1-E02) into a 4× demagnifier consisting of a 180 mm focal length achromat (Thorlabs AC508-180-A-ML) and microscope objective (Olympus PLN4X) to strike a galvo mirror (Scanlab Dynaxis 3S). The demagnification ratio must be chosen to minimize the beam diameters on the galvo mirror, within the limits set by lens working distances and the need to fit the resulting foci on the mirror array. Because the beam diameters on the galvo mirror are on the order of one hundred microns, when projected through a 200 mm focal length lens (ThorLabs ACT508-200-A-ML) onto the mirror array, paradoxically the size of the resulting ‘focus’ is significantly larger than that of the ‘collimated beam’ reflected from the galvo mirror. The focus diameter *d* can be estimated from the Rayleigh criterion *d* ≈ 0.61λ*f*/*D*, where λ is the wavelength, *f* is the focal length of the lens and *D* is the aforementioned beam diameter. In practice, 4× demagnification is acceptable.

The beams are focused onto a mirror array by an achromatic lens (Thorlabs ACT508-200-A-ML placed in a telecentric scanning configuration). This mirror array consists of nine custom-made kinematic mounts with three fine adjusters (Thorlabs F2ES8) per mount, using superelastic nitinol wire as the source of spring tension. Each platform is tapped with an M2 hole in the middle, within which a matching set screw is located; a mirror (Edmund Optics 87-366) is glued on the screw head using thread locking glue. The axial position of the mirrors can therefore be adjusted using the set screw, which is important when using a diode laser with coherence length as short as a few tens of microns. For our prototype, we use a Diode-Pumped Solid State (DPSS) laser with a more practical ∼2 mm coherence length. Thus, the mirrors are coarsely aligned to have a 1 mm offset along the optical axis between each row to compensate for the path-length differences between the three laser beams. Such path-length differences are introduced in the three-way beamsplitter by the half wave plates; the top and middle beams travel through 2 and 1 more half wave plates than the bottom beam, respectively. An exploded diagram of the mirror array can be seen in Supplementary Figure S4.

Optionally a quarter wave plate cut from a polymer sheet (Meadowlark BQ-200 × 200-0473) can be placed in the correct orientation in front of each mirror to control the linear polarization. One of the challenges for SIM is that at high numerical aperture, efficient interference between two beams requires both to have s-polarization due to the vectorial nature of light. In our case, the laser beam was introduced with a horizontal polarization. For the 6 beams reflecting off the side columns of the mirror array, the majority of the power (75%) is s-polarized and therefore a high contrast interference contrast can be obtained. However, the beams reflecting off the middle column of the mirror array have p-polarization. To rotate polarization, an approximately 5 × 20 mm^2^ strip was cut from the quarter-wave plate sheet with a 45° angle from the fast axis, and placed in a custom-made holder in front of the middle column of the mirror array. As the laser beams pass through the quarter-wave plate twice before and after reflecting off the mirrors, the polarization was changed from horizontal to circular and then to vertical, i.e., s-polarization when the beams reach the microscope.

The kinematic mounts are adjusted to steer the beam from each mirror so that it passes through the center of the entrance pupil of a desired lens in a miniature lens array. This lens array consists of 7 achromatic lenses (Edmund Optics 63-714, 4 mm diameter, 6 mm focal length) in a hexagonally close-packed array. These bring the light from each beam to a tight focus at the back aperture of a well-corrected large-aperture lens (Thorlabs TTL200MP), forming an interference pattern at its image plane. The interference fringes are projected onto the image plane of a microscope, which relays the pattern onto the sample.

### SIM Setup

To incorporate SWIFT into a SIM microscope, the interference fringes are placed at the image plane and relayed to the sample via the tube lens (Thorlabs TTL200MP), dichroic mirror (Semrock Di03-R473-T1-25 × 36, specified <1λ peak-to-valley wavefront error) and microscope objective (100× 1.5 NA TIRF Olympus UPLAPO100XOHR). All components are mounted in an Olympus IX73 inverted microscope frame, with a custom-made deck insert. For 3D SIM measurements, the focus position was controlled automatically using a stepper motor (Prior Scientific PS3H122R with Proscan III controller) attached to the fine focusing knob of the microscope.

To ensure that the image of the foci lies just within the back aperture of the objective, a Galilean beam expander combining two lenses (Newport KPC067 and Thorlabs LA1727-AB) is applied between the two TTL200MP lenses to adjust the effective period of the fringes. Images of the sample are projected back through the objective and dichroic, through an emission filter (Semrock BLP01-473R-25) and projected onto a high-speed high-performance camera (Photometrics Kinetix) with the built-in tube lens of the microscope frame.

### Alignment Procedure

To align the SWIFT modules, light is first aligned through the beamsplitters. Two mirrors are used to steer the laser to the axis defined by the beamsplitter centers; the half-wave plates are rotated to provide roughly equal light intensity from each beamsplitter. The beamsplitters are rotated and tilted to coarsely align each beam onto its respective pick-off mirror. The middle beam is used as a reference to align the rest of the system; its beam strikes the D-mirror, which is adjusted so that the beam hits the center mirror of the kinematic mirror array when the galvo is in a neutral position. The tilt and rotation of the remaining beams can then be adjusted using their corresponding pick-off mirrors so that the beams strike the mirrors above and below the center.

Once the beams are centered on the correct mirrors in the array, the kinematic adjusters behind the mirrors are used to back-project the light through the optical system to the desired miniature lens, and finally to the microscope. Custom-made beam blockers are placed in front of the mirror array to allow only one beam to pass for individual adjustment of the mirrors. A layer of fluorescent nanoparticles is used to observe the illumination pattern. The kinematic adjusters are fine-tuned so that the center of the field of view is evenly illuminated. The galvo is then rotated to a positive (or negative) angle so that the beams hit the mirrors on the left (or right) column. The kinematic adjustments are repeated for the side mirrors. After the above procedure, the system is ready for 2-beam SIM with the middle beam blocked by the custom-made beam blocker.

In order to generate accurate 3-beam interference for 3D-SIM, the 2 side laser beams must follow a symmetrical path about the center beam. This means that all 3 beams need to exit the beam splitter in parallel and the center beam must pass the center mini-lens. Any asymmetry in the beam direction can cause a “beating” effect in the resulting interference pattern, significantly degrading the reconstructed images. Therefore, the angle of the laser beams needs to be fine-tuned based on the generated interference pattern. This is an iterative process as the kinematic mirrors need to be adjusted to optimize the observed pattern. The quality of the interference pattern can be directly observed from the image of the nanoparticles. A good 3-beam interference pattern should have even fringe contrast across the field of view while an asymmetric interference pattern would exhibit alternating bands of fringes and nodes.

Finally, the galvo is calibrated to obtain the voltage setting for the required phase shifts. To do so, the galvo is scanned in fine steps near the 3 angles corresponding to the 3 mirror columns, and images of fluorescent nanoparticles taken. The corresponding phase shifts are estimated using the homemade SIM reconstruction algorithm; the results exhibit an approximately linear dependence on the voltage. The voltage settings are subsequently chosen to achieve even phase steps of 2π/3 and 2π/5 for 2D and 3D SIM, respectively.

### Sample Preparation

The fluorescent nanoparticles were prepared in two ways. For switching speed test, the suspension of nanoparticles (Fluoresbrite YG Carboxylate microspheres 0.2 μm) was diluted, dropped on to a coverslip and spread using the pipet tip before being allowed to dry. Subsequently, a few drops of optical adhesive (Norland 81, *n* = 1.56) were dropped onto the nanoparticles and then covered by a microscope slide, with another 2 slides as spacers between the coverslip and the covering slide, resulting in a 1 mm layer of adhesive on the beads after being cured using ultraviolet exposure. The nanoparticles were then imaged through the coverslip.

For 2D TIRF SIM, the suspension of nanoparticles (Fluoresbrite YG Carboxylate microspheres 0.1 μm) was diluted, dropped on to a 35 mm glass-bottomed confocal dish before let dry. Afterward, a small drop of optical adhesive (NOA 1348, *n* = 1.348) were dropped onto the nanoparticles and cured under a small piece of coverslip to reduce oxygen inhibition. After removing the coverslip and uncured adhesive, the adhesive layer serves as a protection for the nanoparticles. A few drops of water were then added to the dish to create an aqueous environment for TIRF imaging.

U2OS FlipIn Trex cells were cultured in DMEM (Corning) supplemented with 10% fetal bovine serum (Gibco) and 1% penicillin–streptomycin (Gibco) at 37 °C with 5% CO_2_. Cells were trypsinised and plated on #1.5 coverslips coated with fibronectin (Sigma, F1141, 50 μg/mL in PBS), for 2 h at 37 °C in DMEM-10% serum. Cells were screened for mycoplasma using MycoAlert Mycoplasma Detection Kit (Lonza). The medium was removed and the cells were washed twice with warm PBS and fixed with 4% paraformaldyhde. After a further two PBS washes, 100 μL of 1 μL in 1 mL phalloidin-iFluor 488 (Abcam) with 1% BSA was added and left for 90 min. Cells were then washed again twice with PBS and mounted onto slides using Fluoromount-G (Invitrogen).

## Results and Discussion

### Design

SWIFT consists of five main parts: a *beamsplitter unit*, a *4f demagnifier*, a *galvo mirror assembly*, a *miniature mirror array*, and finally a *miniature lens array*, as illustrated in Figure 2. Beamsplitter units can be constructed from a diffractive element, as is typically done for SIM, but to achieve achromaticity and minimize loss of light we designed a beamsplitter unit using a series of achromatic half-wave plates and polarizing beamsplitters (see Figure 2 bottom right, and Supplementary Figure S2). The power of each beam is controlled by the half-wave plates, which change the beam’s polarization and thus the fraction reflected from the beamsplitters. A subsequent set of pick-off mirrors allow the beams to be steered independently while passing close to each other. The unit was assembled on a custom-machined baseplate using small FiberBench components for reasons of stability and cost-efficiency.

After the beamsplitter unit, the beams strike a pick-off mirror and are imaged onto the galvo using a telecentric 4f optical system with a substantial demagnification (4× in our case). The galvo reflects the beams to their respective mirrors, addressing a column of mirrors at a time. Each mirror is mounted on its own kinematic platform, with tip-tilt adjustment and a screw thread for axial displacement (for the purpose of matching path-lengths, see Supplementary Figure S4. The mirrors reflect the light back toward the galvo and then to the miniature lens array. Because the galvo surface is conjugate to the lens array, the tilt of each mirror (controlled by its kinematic mount) determines which lens the beam passes through. Attenuation and polarization control can be performed on a per-mirror basis by placing a neutral density filter and a polymer quarter-wave plate in front; the light passes the wave plate twice, and thus it acts as a half-wave plate, rotation of which controls the linear polarization axis orientation.

The focus of the lens array is at the back focal plane of a large lens; therefore, each focus is converted to an angled plane wave, all of which interfere at the front focal plane to produce the desired interference pattern. Since the coarse position of the galvo determines the column of the mirrors addressed by the beams, switching between columns allows different interference patterns to be created, with arbitrary combinations of angle, intensity and polarization, albeit with the requirement that these parameters must be determined ahead of time. Furthermore, when moving the galvo a smaller distance, such that beams still address the same column of mirrors, the path length of one beam can be changed relative to another. Consequently, a phase shift is applied to the interference pattern, without changing the structure of the pattern itself. The ratio of the phase shift to the change in the galvo angle is proportional to the horizontal distance between the relevant lenses.

The galvo stability requirements are dictated by the size of the lens array and the aforementioned demagnification ratio; the galvo is conjugate to the lens array and therefore to maintain a desired phase relationship between the outermost beams, the galvo must not jitter excessively. For example, to maintain a λ/10 stability at 488 nm, with a 2 mm separation between the beams on the galvo, the galvo should be stable to arctan­(48.8 nm/2 mm) ≈ 24 μrad. Fortunately, the Dynaxis 3s galvo selected for the prototype boasts better than 1 μrad repeatability.

### Performance

To assess the interference patterns generated by SWIFT, we imaged the center of the pattern using a separate home-built 4× microscope. Assessments were performed on three types of interference patterns: a two-beam interference pattern, a hexagonal three-beam pattern and a linear three-beam pattern. Figure 3a,b show a measured two-beam interference pattern generated by overlapping the top and bottom beams originating from the positions on the back aperture highlighted in the inset. By fitting the image to a 2D sinusoidal function with an offset, we obtain a fringe visibility (*V* = (*I*_max_ – *I*_min_)/(*I*_max_ + *I*_min_)) of 0.92. Similar results can be obtained from the other two orientations. A hexagonal pattern was generated by overlapping three beams through the outer lenses, as shown in Figure 3c,d. As a horizontal offset between the lenses is necessary for the phase shift, the lens array in this case was rotated by 90° from the two-beam interference configuration. Finally, Figure 3e,f show the three-beam interference pattern commonly used for 3D SIM.^5^ Because this pattern is periodic in z as well, we also measured the intensity distribution as a function of axial position in Figure 3g,h. To do so, the objective of the home-built microscope was moved along the optical axis using a high-precision motorized translation stage. The measured images show high-contrast fringes similar to the expected three-beam interference pattern, except for a shift in the vertical direction in Figure 3g which is attributed to misalignment between the SWIFT optical axis and the direction (*z* axis) along which the camera was moved.

**Figure 3:**
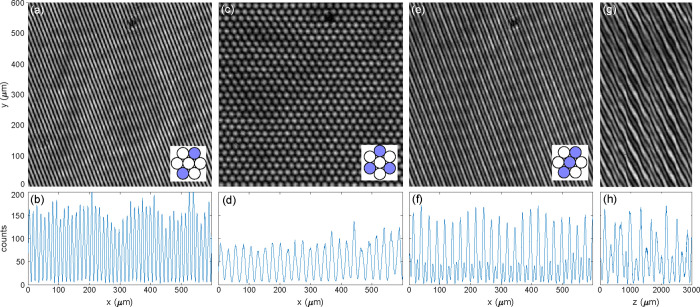
Centre interference patterns near an intermediate image plane measured using a 4 × C microscope. (a) Two-beam interference pattern, (c,d) Hexagonal interference pattern. (e–h) Three-beam interference pattern. (g) is obtained by combining y-cuts in the middle of 601 camera images of the three-beam interference pattern at different axial positions. (b,d,f,h) are horizontal cuts of (a,c,e,g) at *y* = 300 μm. The insets illustrate the orientation of the lens array, with the lenses used highlighted in blue.

We evaluated the speed at which the interference pattern could be changed by recording high-frame-rate fluorescence images at 3252 fps. The interference patterns were projected onto a 100× microscope (which will be used for SIM measurements in the following section), ultimately onto a layer of 200 nm fluorescent beads, which was used to reveal the illumination profile. For high frame rate, the camera was set to a low dynamic range mode (8 bits) with a Region of Interest (ROI) of 65 μm × 13 μm in the center of the sensor. In order to resolve the pattern with the microscope, we use the interference between the top-right and middle beams using the lenses highlighted in Figure 4. The galvo was rotated back and forth at 300 Hz to switch between the three orientations, with five phase steps per orientation. The phase steps were set to be slightly larger than the steps used for 2-beam and 3-beam SIM, to provide a rigorous test.

**Figure 4:**
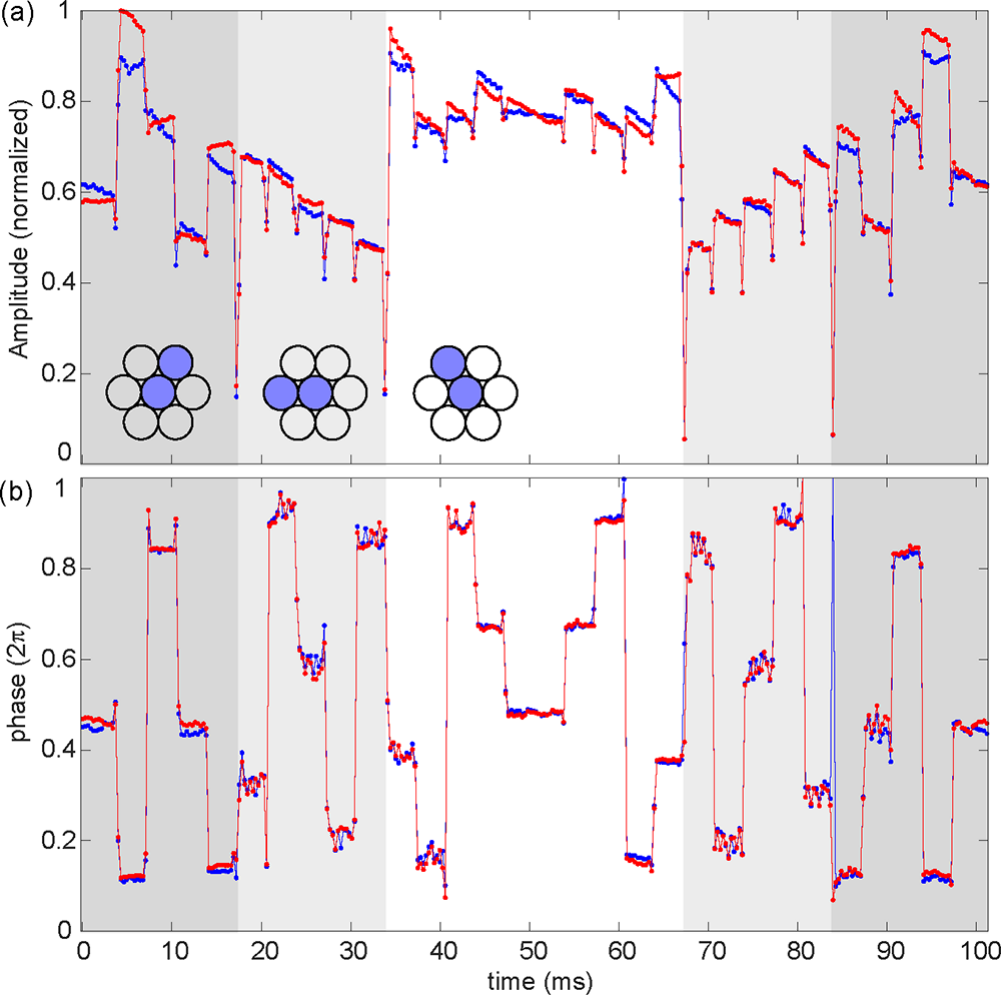
Stability and switching speed of interference patterns. Sequences of fluorescence images of the interference patterns were taken at 3252 fps while the galvo was set to a list of 15 positions, corresponding to 3 different resulting interference fringe orientations with 5 positions for each orientation. The galvo stayed for 1/300 s at each position and rotated back and forth. The correlation between the Fourier transform of each image and the Fourier transform of the average of all images are calculated to obtain the (a) amplitudes and (b) phases of the maximum correlation points for each image. Results from two rotation periods (red and blue) with the same galvo setting are compared. The background colors indicate the three different fringe orientations and insets illustrate the corresponding mini lens profile. The phases at the plateaus of the two image sequences match with fluctuations of a few percent of 2π. Laser power: 100 mW. Exposure time: 300 μs.

To examine the quality of the interference patterns, we correlated the Fourier transform of each image with the average of all images. The amplitude, phase and position of the maximum of the correlation reflect the visibility, phase and orientation of the interference patterns, respectively. A similar process can be used to determine parameters for SIM reconstruction. When the galvo reached a stable state, the maximum amplitude of the correlation function remained at a plateau. When the galvo switched, the amplitude of the correlation dropped significantly during the transition frame(s), with concomitant uncertainty in the phase value. Figure 4 shows the maximum amplitude of the correlation and the corresponding phase obtained from two sequences of images with the galvo switching between 15 different positions, i.e., fifteen interference patterns. The phase transitions commonly occurred within one frame (only one frame had reduced correlation for each transition), while the transition between different angles required as many as three frames, i.e., 0.9 ms. As the galvo rotation for phase shift was less than 1% of the rotation for the orientation transition, the results suggest that the actual phase transition time could be as little as 10 μs. The experiment was performed twice, with the phase of the two image sequences (shown in blue and red) matching well. This indicates that the phase is repeatable and stable, with fluctuations of just a few percent of the full 2π range, albeit slightly higher than the theoretical stability of the galvo. We attribute this small decrease in performance to electronic noise in the galvo driver. Environmental factors such as temperature change and vibration may cause further phase drift over longer time scale (a few percent over minutes, or multiple tens of percent overnight, see Supplementary Figure S5). This is not an issue as long as the phase is estimated per image during reconstruction. Despite the many precision elements that need to be aligned, the alignment can remain stable over days.

### Structured Illumination Microscopy (SIM)

The utility of SWIFT is demonstrated for both 2D TIRF-SIM and 3D SIM. For 2D TIRF-SIM, we illuminated with just two beams; the beamsplitter unit was adjusted to reduce the power of the middle beam to near-zero, and a custom-made beam blocker, placed in front of the middle row of the mirror array, was used to eliminate any residual intensity.

The imaging speed for 2D SIM is commonly limited by the switching time between different imaging patterns, the minimum exposure time to obtain enough fluorescent signal, and the speed of the camera. SWIFT is capable of high speed SIM imaging, with transition speed up to a theoretical 100,000 frames per second for phase shift and 1000 frames per second for angle change according to the performance test on 65 μm × 13 μm ROI, assuming perfect synchronization between the mirror and the camera. To demonstrate this capability, we imaged a layer of 100 nm fluorescent nanoparticles sealed under a layer of UV-cured adhesive (NOA 1348, *n* = 1.348) and covered by water. Considering the speed limit of our camera for such ROI and high data dynamic range (16 bit), we chose an exposure time of 1 ms and a raw frame rate of 980 fps. The galvo signal was synchronized with the camera trigger to repeatedly set the galvo to one of 9 positions, specifically 3 phase steps for 3 orientations. The rotation direction of the galvo alternated between consecutive SIM imaging cycles, so that its position was unchanged during the first frame of each cycle. To accommodate the longer switching time (approximately 1 ms) between different orientations, the galvo was programmed to remain for two frames in the first phase of the second and third orientations. Consequently, each cycle took 11 raw camera frames, yielding a final imaging rate of 89 fps. With the first frames after the orientation change discarded, 9 out of the 11 frames were processed using a homemade MATLAB program based on open source algorithms^16,17^ to produce a super-resolution image. A theoretical PSF assuming NA = 1.333 was used for reconstruction. Figure 5 shows an example of a reconstructed image along with the corresponding wide-field image with the theoretical PSF deconvolved. In addition, for the sake of completeness, the same data was reconstructed using FairSIM^18^ and HiFi SIM;^19^ the results can be seen in Supplementary Figure S6. The SIM image reconstructed using our code shows a clear improvement in resolution which is sufficient to resolve beads with a separation of 100 nm, such as the two marked by arrows in Figure 5d. Here the parameters used for reconstruction (interference pattern angle, spatial frequency and phases) were estimated from the images using a non-TIRF parameter estimation algorithm. This was enabled by the higher refractive index of the fluorescent nanoparticles, which extends the emission OTF beyond the theoretical NA of 1.333. Meanwhile, the scattering of nanoparticles changed the illumination field and emission OTF, resulting in artifacts in the reconstructed image as commonly observed in TIRF SIM. By calculating the Fourier ring correlation between images reconstructed from two consecutive imaging cycles, we found the resolution to be 99 nm.

**Figure 5:**
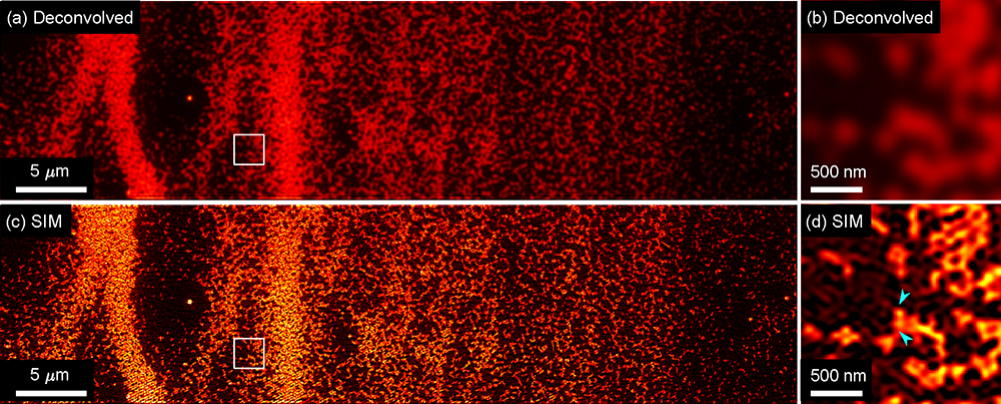
TIRF images of 100 nm fluorescent beads. (a) Widefield image obtained by averaging 9 SIM frames and deconvolving the theoretical PSF and (b) a zoomed section. (c) Corresponding reconstructed SIM image and (d) a zoomed section at the same position as (b). The arrows in (d) point to two beads with a vertical peak separation of 100 nm. Exposure time: 1 ms per frame, final super-resolved frame rate: 89 fps. Laser power: 300 mW.

It was noted during the previous experiments that the achievable super-resolved field of view was much less than anticipated, owing to field-dependent aberrations. To characterize this, a sample of 200 nm fluorescent beads embedded in Norland Optical Adhesive NOA81 was imaged over the whole field of view. The image quality at various distances from the optical axis could then be assessed. The results (see Supplementary Figure S8) show a clear trend of increased artifact severity and poorer image quality with distance from the image center; super-resolved information can be seen up to ∼40 μm away from the image center, for a field of view of ∼80 μm, compared to the objective’s 220 μm field of view. Further analysis may be found in Section 6 of the Supporting Information.

Next, we applied 3D SIM imaging to iFluor 488 phalloidin-stained U-2 OS cells with 3-beam illumination. The beamsplitter unit was adjusted to optimize interference contrast. To obtain steps the *z* direction, we moved the objective using a stepper motor focus drive with a step size of approximately 130 nm. At each position, 15 frames were taken with 3 orientations and 5 phase steps. Figure 6 shows a comparison of a deconvolved widefield image and a SIM image of the same location. The SIM image shows clearly improved resolution in both lateral and axial directions. To further assess the lateral resolution, we split the raw image stack into subsets with odd and even z steps. A superresolution 3D image was reconstructed independently from each subset with double *z* step size. By calculating the Fourier ring correlation between two lateral cuts at about the same position as Figure 6b, we found the lateral resolution to be 138 nm.

**Figure 6:**
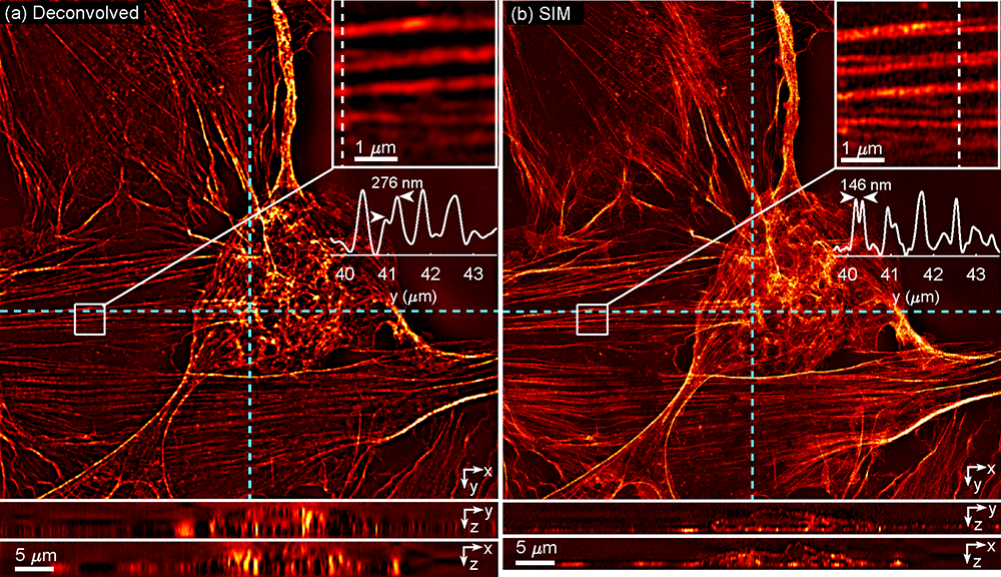
3D SIM imaging of U-2 OS cells stained with iFluor 488 phalloidin. (a) Sections of a 3D widefield image after deconvolving the theoretical PSF. YZ and XZ projections below correspond to the locations marked with the cyan dotted lines. An inset image shows the diffraction-limited imaging performance in a selected region with a cut along the white dotted line. (b) Sections of a reconstructed 3D SIM image. YZ, XZ projections and inset image are taken from the same locations as (a), with resolution improved in both the lateral and axial directions. Exposure time: 199 ms per frame. Laser power: 20 mW.

### Discussion

The SWIFT concept contains a number of subtleties that warrant discussion. The first is the 4× demagnification between the lens array and the galvo, which was chosen as a trade-off between the system stability and practicality. Since the galvo and lens array are conjugate to each other, a stronger demagnification will make for a smaller image on the galvo and thus increased phase stability against the jitter of the galvo; this comes at the cost of larger spots on the miniature mirror array, potentially requiring the mirror array to be impractically large.

Next is the choice of parameters for the lens array, the purpose of which is to maintain a small spot size at the back aperture of the final lens, thereby increasing the diameter of the beams at the plane of the interference pattern and thus increasing the patterned area to practical levels. There are no theoretical restrictions on the location, focal length or diameter of the miniature lenses, since a decrease in diameter can be compensated by a proportional reduction in focal length. However, on a practical level this reduction in lens diameter cannot be applied excessively when considering the stability of the kinematic mounts in the mirror array. These mounts determine the positions where the beams strike the lens array; smaller lenses would require more accurate kinematic mount alignment and greater stability, as well as requiring an even smaller beam size than the lens diameter. It should be noted that the lens array needs not to be in any regular pattern; arbitrary patterns can be created by positioning individual lenses, or by fabricating them in one piece using 3D-printing,^20^ single-point diamond turning or photolithography.^21^

SWIFT compares favorably with conventional methods of pattern projection. The use of a single galvo mirror to control all parameters makes it possible to reach submillisecond pattern switching speeds that conventional moving gratings and most commercial off-the-shelf SLMs cannot match. Only advanced display technologies like DMDs and ferroelectric SLMs can compete in this regard, but are chromatic (requiring reconfiguration for each different wavelength), highly inefficient due to substantial power loss to unwanted diffraction orders, and size-limited due to the finite display resolution. As an illustration, the field of view of an Olympus UPLAPO60XOHR microscope objective is specified as 22 mm/60 = 366 μm diameter. The highest resolution DMD currently available is the Texas Instruments DLP991U, at 4096 × 2176 pixel resolution. Since the SIM fringe pattern would have a period of λ/2NA where λ is the wavelength and NA is the numerical aperture on the objective, and because the DMD must Nyquist-sample the fringes, the maximum illuminated area would only be 323 μm × 172 μm, less than 53% of the total field of view.

In contrast to these techniques, SWIFT is achromatic and power-efficient. The only theoretical limitation on field of view is power density (since the illumination intensity pattern is just the image of the laser on the back aperture of the miniature lenses, magnified by the ratio of focal lengths of the miniature lens and the “scan lens,” either of which can be varied almost arbitrarily.) As noted in the results and Supporting Information, however, aberrations in the imaging system can limit the field of view to substantially below the objective’s specified value. Turning to achromaticity, while we have not demonstrated the use of a second laser due to funding limitations, there are no wavelength-dependent components in the system and no configuration changes are required to change wavelength, which is important when performing high-speed multicolour imaging. Furthermore, SWIFT exhibits no higher order diffraction, does not require tight focusing at any point, and all the optical surfaces insensitive to laser damage. This makes it more desirable for high laser power applications than fiber-based solutions.^22^ Finally, SWIFT’s ability to control polarization by simply adding quarter-waveplates is another advantage over DMDs and SLMs, which must either use an electro-optic modulator to rotate the plane of polarization of all beams, or use a so-called “pizza polarizer” consisting of individual waveplates placed conjugate to the entrance pupil of the objective lens. This latter solution requires a particular fixed polarization for each beam, which is problematic in 3D SIM as the center beam must change polarization to match the outermost azimuthally polarized beams.

It is worth noting that this is an initial prototype and that there is still room for improvement in the design. The illuminated area on the galvo is very small, implying that further improvements in projection speed can be achieved simply by using a lighter galvo mirror, or even a miniaturized MEMS design. The size of the kinematic mirror array was originally chosen to fit within the field of view of a 2″ diameter achromat, but readers may prefer to use a large-format achromat or parabolic mirror; the mirror array can then be made larger, using off-the-shelf kinematic mounts, which in turn improves stability, ease of alignment, and cost. Alternatively, for situations where more fringe patterns must be employed, the larger format lens can be combined with the existing 7 mm × 7 mm footprint kinematic mirrors; a 4″ diameter lens can support 14 sets of three mirrors, but if the galvo is replaced with a galvo pair or 2D MEMS mirror, 42 three-elements-per-column mirror configurations can be supported. The miniature lens assembly can be made more flexible by use of a commercial hexagonal microlens array, broadening the range of interference patterns that can be created. Alternatively, the miniature lenses and the mirror array could be fabricated monolithically for use in shared facilities and other high-usage environments where stability is at a premium. Finally, the system is achromatic, so it is able to incorporate many more laser wavelengths simultaneously.

## Conclusions

In summary, we have presented SWIFT, a system for creating arbitrary interference patterns using a single moving part, and used it to perform 2D as well as 3D SIM. Data were taken at high speed (up to 980 fps raw frame rate), which is important for capturing super-resolved dynamic biological events.^23^ Despite just being a prototype, performance parameters were competitive with the state of the art in SIM instrument design.

## Supplementary Material



## Data Availability

A CAD model of the instrument, the data sets generated and/or analyzed during the current study are available in the zenodo repository, 10.5281/zenodo.15360998.

## Notes

Design and postprocessing details for synthesis of arbitrary interference patterns using a single galvanometric mirror (PDF)
