# Supplementary material for: Synthesis of Arbitrary Interference Patterns Using a Single Galvanometric Mirror and Its Application to Structured Illumination Microscopy
Source: ACS Photonics. 2025 Jun 19;12(7):3635–43. doi: 10.1021/acsphotonics.5c00516 (PMC12272674; doi:10.1021/acsphotonics.5c00516)
Supplement: Supplementary file 1 [file ph5c00516_si_001.pdf]

Supporting information: Synthesis of arbitrary interference patterns using a single galvanometric mirror, and its application to Structured Illumination Microscopy

Ke Guo,[†] Abderrahim Boualam,[†] James D Manton,[‡] and Christopher J Rowlands[*,†]

†*Department of Bioengineering, Imperial College London, Exhibition Road, South Kensington, SW7 2AZ, United Kingdom*

‡*MRC Laboratory of Molecular Biology, Francis Crick Avenue, Cambridge, CB2 0QH, United Kingdom*

E-mail: c.rowlands@imperial.ac.uk

1 Adjusting focal separation

The miniature lens array determines the angle of the interfering plane waves, but in some cases it is desirable to tune these angles by changing the magnification of the foci created by the lens array. In SIM for example, the image of these foci should lie just within the back aperture of the microscope objective lens. To tune the spacing of the foci, a lens system can be placed between the two lenses that relay the foci to the objective back aperture. Three example configurations (for spacings of 4.6 mm, 5 mm and 7 mm) are given in Supplementary Table S1-S3, and their layouts can be seen in Supplementary Figure S1.

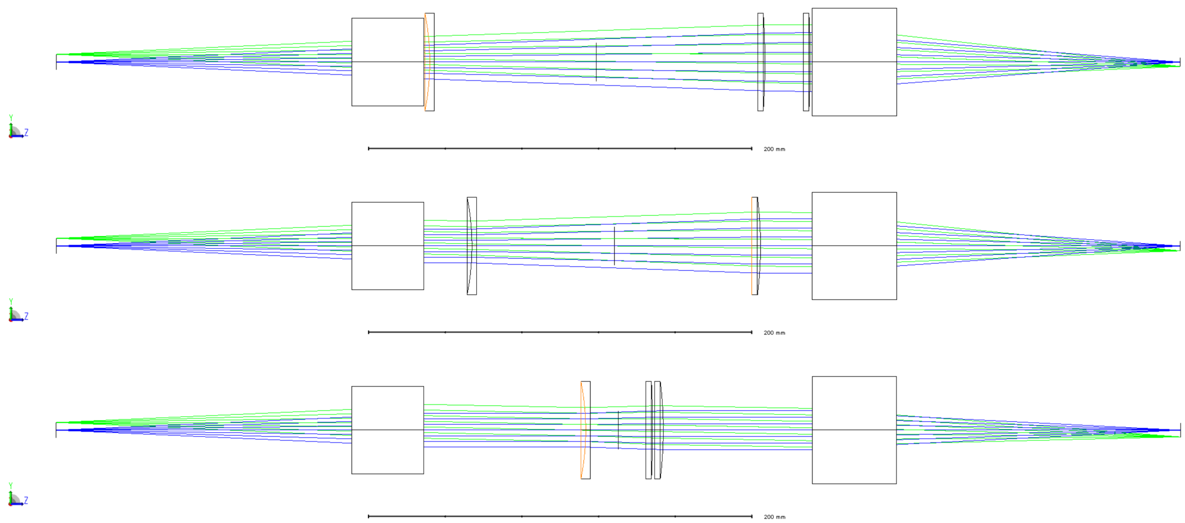

Figure S1: Layouts for adjusting focal separation. Top: 4.6mm focus separation. Middle: 5mm focus separation. Bottom: 7mm focus separation. Total track length is 432.3mm in all cases.

Table S1: 4.6mm focal separation

Lens	*Distance to next lens surface*
Miniature lens focus	154.1mm
ThorLabs TTL200MP	3.0mm
Newport KPC067	168.6mm
ThorLabs LA1727-A	20.0mm
ThorLabs LA1779-A	1.0mm
ThorLabs TTL200MP	148.0mm
Objective back aperture	

Table S2: 5mm focal separation

Lens	*Distance to next lens surface*
Miniature lens focus	154.1mm
ThorLabs TTL200MP	25.0mm
Newport KPC067	143.5mm
ThorLabs LA1725-A	26.9mm
ThorLabs TTL200MP	148.0mm
Objective back aperture	

Table S3: 7mm focal separation

Lens	*Distance to next lens surface*
Miniature lens focus	154.1mm
ThorLabs TTL200MP	83.1mm
Newport KPC067	28.8mm
ThorLabs LA1779-A	1.0mm
ThorLabs LA1725-A	76.4mm
ThorLabs TTL200MP	148.0mm
Objective back aperture	

2 Custom parts

Several custom parts are needed to assemble SWIFT, primarily because off-the-shelf optomechanics were too bulky. CAD models are all available either upon request or from `https://www.imperial.ac.uk/rowlands-lab/`.

2.1 Beamsplitter fiberbench

The purpose of the fiberbench system is to split the incident beam into three beams of identical polarization but with an arbitrary split of intensity. The system used ThorLabs fiberbench components for reasons of cost and compactness, but required a custom-machined baseplate as suitable commercial alternatives were unavailable. See Supplementary Figure S2.

2.2 Miniature lens and pick-off mirror assembly

The seven miniature lenses are sandwiched between two custom-machined aluminium plates secured inside a lens tube. Before mounting, the lenses were first bundled together using a rubber band to achieve an accurate hexagonal packing. This step should not be neglected, since any inaccuracy in the mounting process can result in asymmetry in the hexagonal arrangement and consequently a beating effect in the 3-beam interference pattern. See Supplementary Figure S3.

2.3 Kinematic mirror array

Because of the limited field of view of the lens between the galvo mirror and the miniature mirrors, the mirror array was custom-built in order to fit a 3×3 mirror array within a 25 mm field of view. Each mirror was mounted on a custom-designed platform that could achieve tip, tilt and piston actuation via a series of set screws; the piston was used for path-length matching in order to optimize the interference contrast and tip-tilt allows each beam to

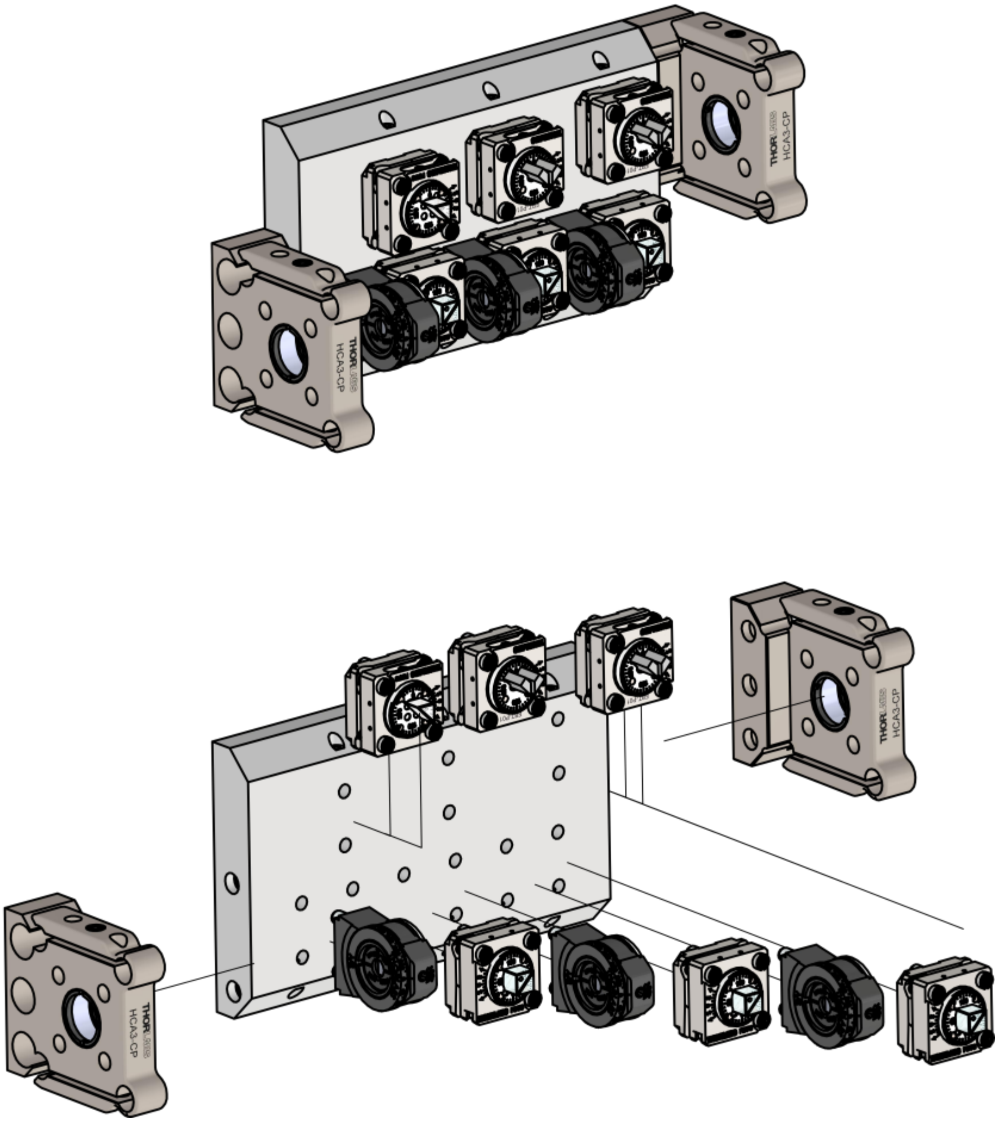

Figure S2: Beamsplitter construction. Top: Laser enters through the bottom cage mount and passes into three sequential pairs of half wave plates and polarizing beamsplitters. These can be tuned to pick off desired fractions of the primary laser beam at each beamsplitter. The pick-off mirror above each beamsplitter allows the beams to be oriented parallel to each other but with a 2 mm offset between them. Bottom: Exploded diagram showing each component and its assembly.

address a particular miniature lens. See Supplementary Figure S4.

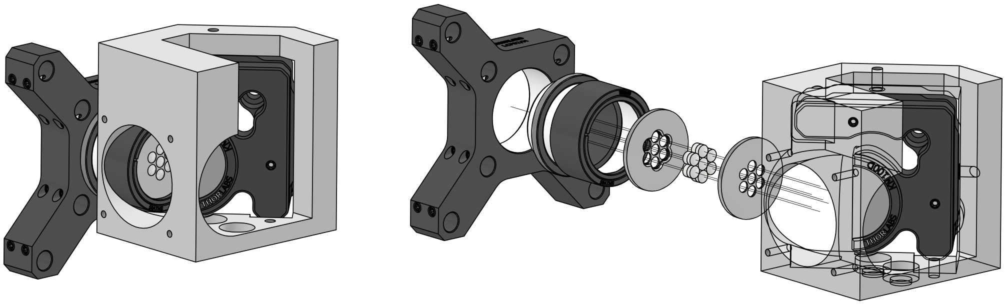

Figure S3: Miniature lens array and pick-off mirror. Left: Light enters from the left; there are standard #4-40 taps for connection to a 30 mm cage system. The light is reflected from a pick-off mirror to the rest of the optical system through the cutout to the right. Returning light illuminates one of seven microlenses, bringing it to a focus. Right: Exploded diagram showing the individual components.

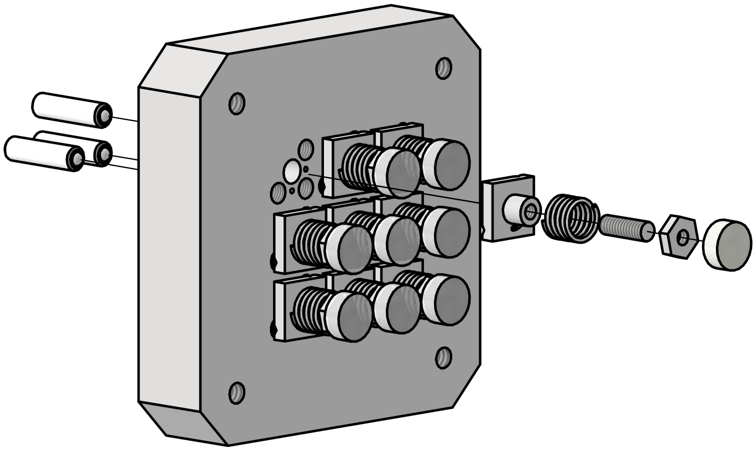

Figure S4: CAD model of the mirror array, with one mirror and its mount shown in exploded layout. Each mirror is mounted on a kinematic platform, which permits adjustment of tip, tilt and axial position. Each custom-machined kinematic platform is secured to the base with superelastic nitinol wire as a spring, with three 0.2 mm pitch set screws to adjust tip, tilt and tension. The mirror itself is mounted on a platform attached to a spring-tensioned set screw which can translate to match the path lengths of each laser beam. The entire assembly is compatible with standard 30 mm cage systems.

3 Pattern stability

The stability of SWIFT was checked by imaging the centre of the two beam interference pattern using the home-built 4× microscope. The phase of the interference pattern was calculated as the phase at the peak of the Fourier transform. Supplementary Figure S5 shows the phase change over three different time scales.

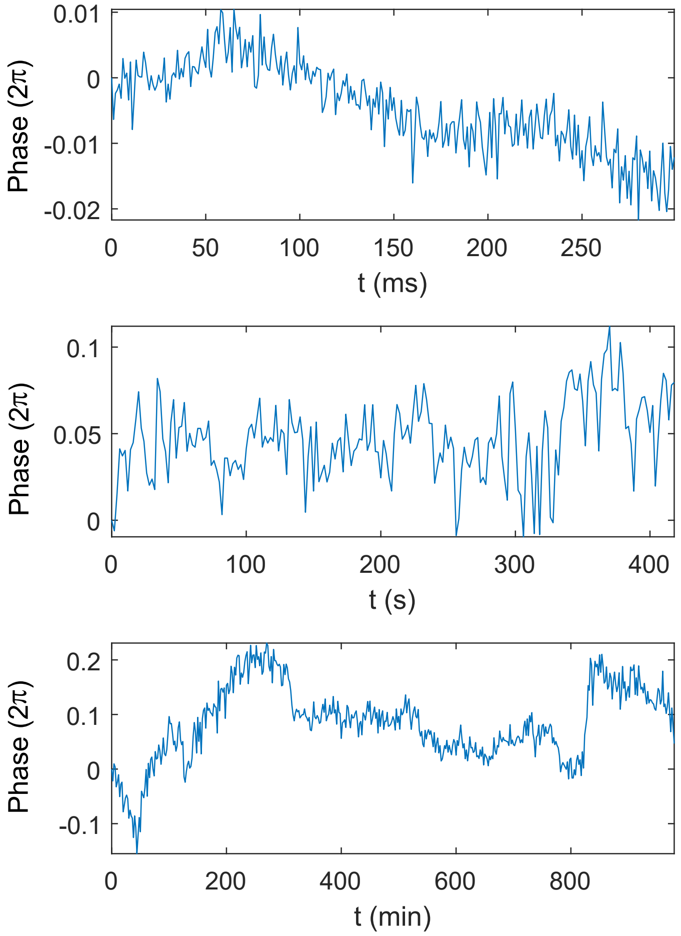

Figure S5: The phase change of the interference pattern over three different time scales. Top: approx. every 1 ms (with frame drop), middle: every 1 s, bottom: every 2 minutes.

4 SIM reconstruction software

The SIM images were reconstructed in the following steps using a home-made MATLAB program partly based on open source algorithms (1, 2).

- **Preprocessing**
  - (Optional) When performing fast 2D-SIM imaging, there can be microscale movement of the sample or camera which degrades the reconstructed image. A sub-pixel image registration algorithm (3) can be used to minimise the influence of such movement where necessary.
  - For 3D-SIM imaging, the raw frames were rearranged to form 15 3D-images with 3 orientation and 5 phases.
  - The 2D or 3D images were normalized to compensate for the different illumination powers of the three orientations. To do so, all images with the same illumination pattern orientation were divided by the same normalization factors which is their average counts.
- **Parameter estimation** The illumination spatial frequencies, the phase shifts and the amplitude of each frequency component were determined *a posteriori* from the pre-processed images independently for the 3 orientations as following.
  - An average of all images were calculated as an estimate of the sample image with homogeneous illumination, i.e. the central frequency component. This is a good estimate for the 2D images. The average 3D image would have an extra modulation in the axial direction, which however does not significantly affect the parameter estimation as the spatial modulations to be estimated are lateral.
  - The illumination spatial frequencies were determined by cross-correlating of Fourier transforms of the pre-processed images and that of the average image. In the 3D case, there are two spatial frequencies. The first order spatial frequencies were

estimated and the second order were calculated by doubling the first order frequencies.

- To estimate the phase shifts, each pre-processed image was considered as a linear combination of 3 components: **(i)** the average image (i.e. the central frequency component), **(ii)** the product of the average image and a 2D cosine pattern with the illumination spatial frequency (i.e. a frequency shifted image component with no phase shift), and **(iii)** the product of the average image and corresponding sine pattern (i.e. a frequency shifted image component with $\pi/2$ phase shift). With all three components estimated from the results of the previous steps, the ratio between the components were determined using linear regression (MATLAB mldivide). The phase shifts of the illumination pattern were calculated from the ratio of the components based on the angle addition theorem.
- Similar calculations were applied to 3D images, except they have five components with two spatial frequencies.

- **Reconstruction**
  - A theoretical OTF was estimated assuming NA = 1.48. The 3D OTF was estimated using the algorithm from (4).
  - The different frequency-shifted image components and the central frequency component were separated based on the estimated parameters using linear regression (MATLAB mldivide).
  - A generalized Wiener filter based on the calculated OTF was applied to all components as well as the corresponding spatial frequency shifts. The amplitudes of the higher frequency components were rescaled so that the average intensity of their overlapping areas in the frequency space matches the average intensity in the same area of the central frequency component. The components were recombined to form a super-resolution image.

– A notch filter (1) was applied to the 3D images to reduce artifacts.

– Images presented in figures were thresholded to further reduce artifacts.

5 Reconstruction using different packages

For the sake of completeness, some of the SIM data (specifically the dataset used for Figure 5) were reconstructed using two prominent open-source software packages, so readers can better compare performance against software that they may be more familiar with. Results can be seen in Figure S6.

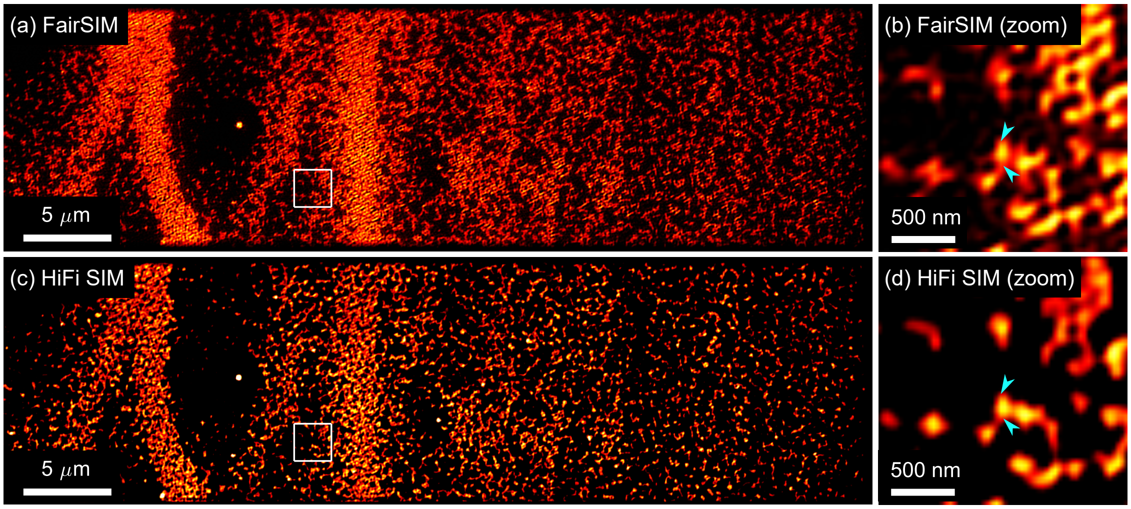

Figure S6: Use of different SIM reconstruction techniques. The same data as that found in Figure 5 is used here. (a) Reconstructed using the ImageJ FairSIM plugin. Parameters were left at default values apart from pixel size (set to 65nm) and OTF attenuation (switched off). (b) Zoomed in section of (a). (c) Reconstruction using HiFi SIM. All parameters were set to their default values with the exception being pixelsize (65nm) and attStrength (set to 0). (d) zoomed in version of (c).

Both algorithms appear to underperform our own code in terms of being able to resolve the fluorescent beads, although HiFi SIM does not appear to suffer from as many artefacts as FairSIM or even (arguably) our code.

6 Field of view (FoV)

While the achievable FoV of SWIFT is primarily limited by the achievable power density, in practice it is also limited by the imaging system and the sample. In this paper, the imaging system consisted of an Olympus UPLAPO100XOHR lens in an unmodified Olympus IX73 frame, with a 25 mm aperture for the fluorescence dichroic and filter. The camera was mounted using a stock Olympus C-mount port adaptor. The sample was imaged through a coverslip or confocal dish. The resulting image exhibited aberrations towards the edge of the FoV, which is commonly observed when imaging through a substrate which has a different refractive index from the immersion medium. Such aberration significantly degrades the performance of SIM and thus limits the FoV. By tuning the correction collar of the objective, we were able to reduce the aberration on the edge and improve the uniformity of the image and the FoV. However, this came at the cost of a small degradation of the resolution in the centre of the image. Figure S7 shows a comparison of measured PSF when using different collar settings. When the correction collar is set to the approximate thickness of the substrate (0.15 μm), the PSF near the edge becomes much less aberrated, although a loss of resolution is still noticeable. A good imaging resolution is also critical for the quality of the SIM reconstructions as it aids in the detection of the fringe spatial frequency, as well as in accurately estimating the SIM parameters. We have not found a significant improvement of the FoV when using different collar settings within a reasonable range. Figure S8 shows an example SIM image taken over the full camera frame, for which the correction collar was optimized for image uniformity. Acceptable super-resolution quality can be observed within a FoV of $\sim$ 80 μm.

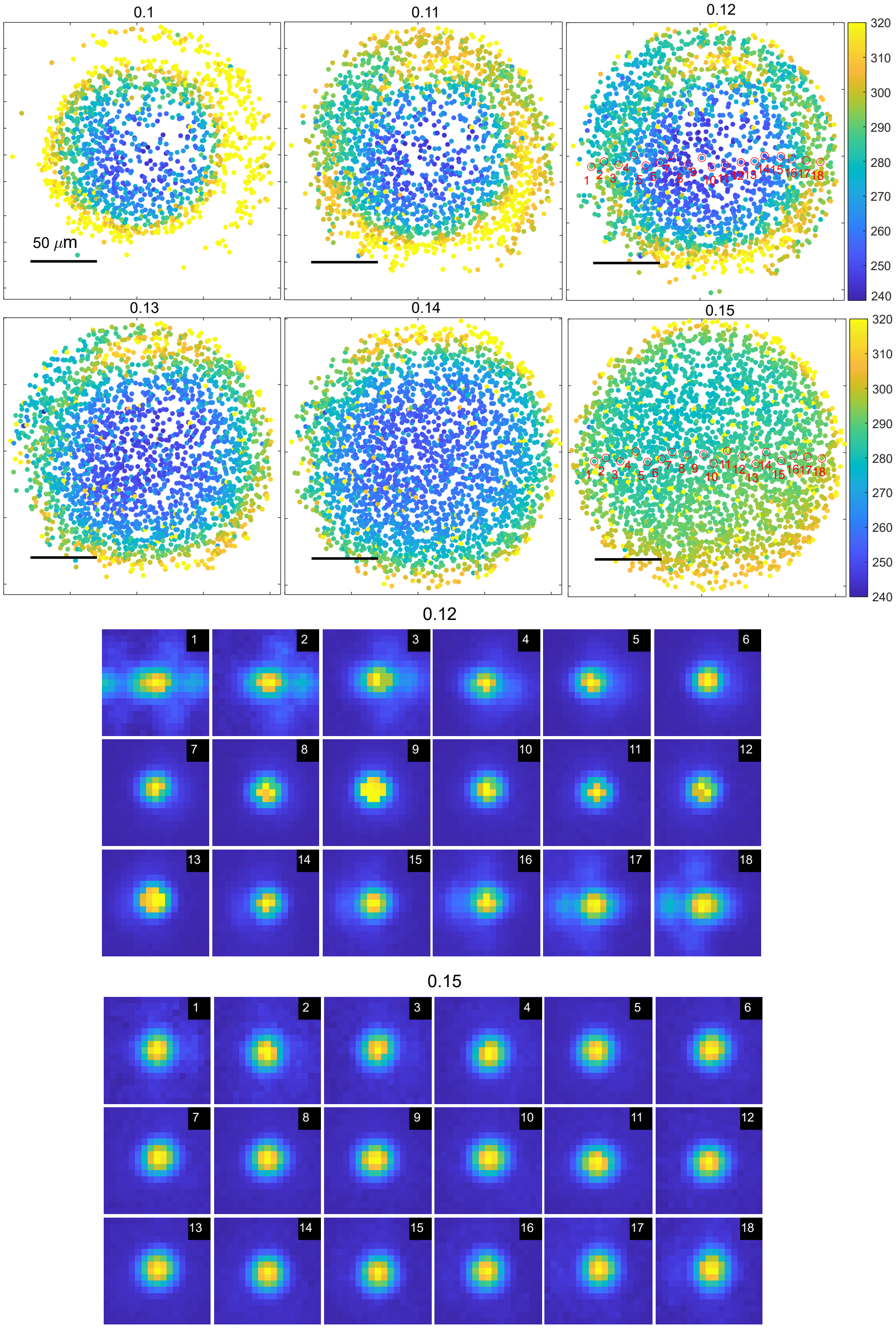

Figure S7: **Resolution as a function of field for different correction collar settings.** The resolution (in nm) was measured using different correction collar settings from 200 nm fluorescent beads (Fluoresbrite YG Carboxylate microspheres 0.2 µm covered by NOA 81 for refractive index matching), and calculated by fitting each bead image with the theoretical PSF formula. For two collar settings 0.12 and 0.15, images of of selected beads (marked and labeled in red) with horizontal distance of ~ 10 μm are shown to illustrate the changes in aberration and resolution.

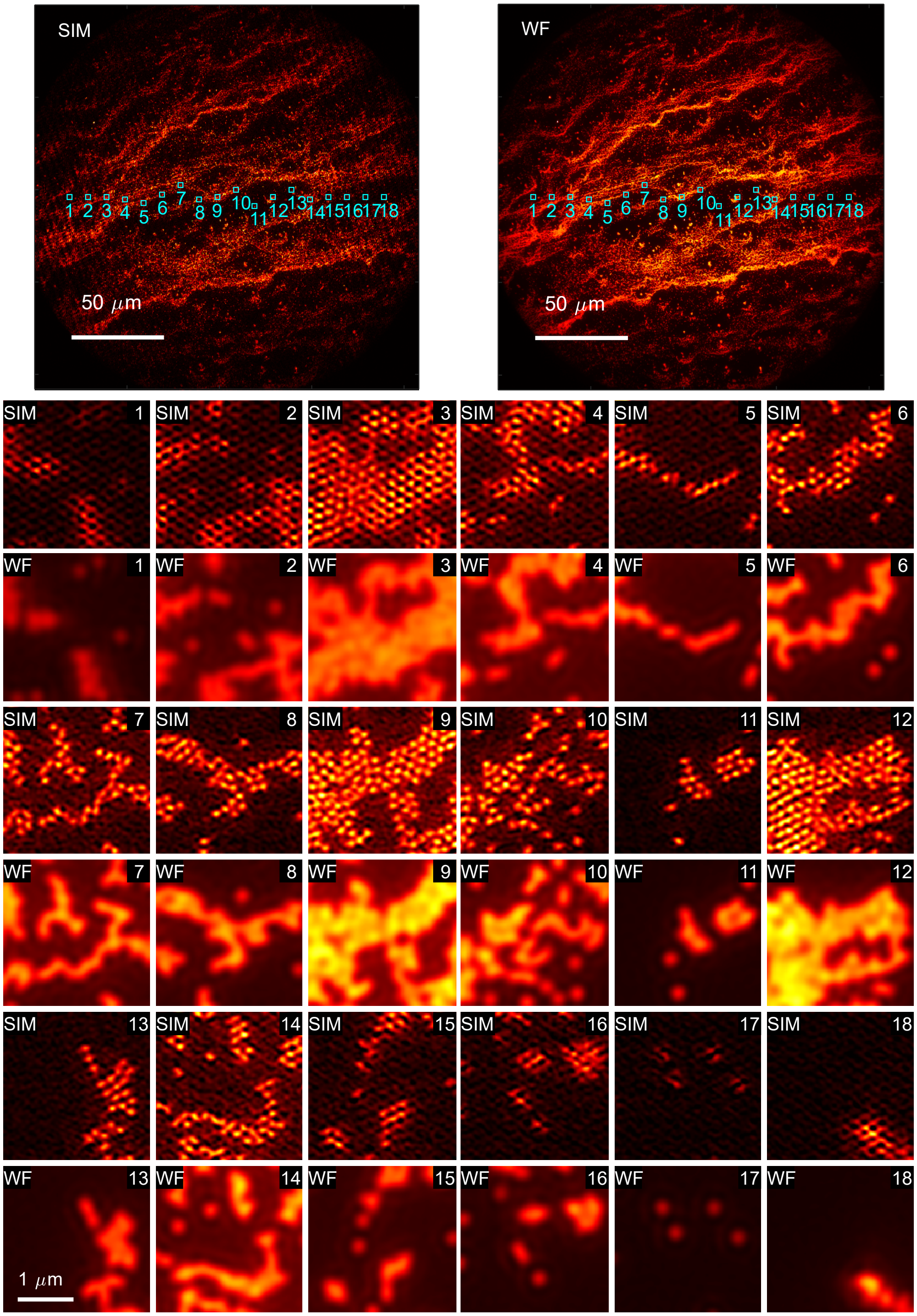

Figure S8: Images of 200 nm fluorescent beads (Fluoresbrite YG Carboxylate microspheres 0.2 μm covered by NOA 81 for refractive index matching) over a large field of view. The super-resolved and widefield data (with PSF deconvolved) can be seen, along with 18 'sub-images' taken from the same location in both images. Each sub-image is spaced at $\sim 10\ \mu m$ intervals horizontally, although they need to be moved slightly to find sparse regions of the sample. The super-resolved image quality appears acceptable from sub-image 6 to 14, implying a super-resolved field of view of around $\sim 80\ \mu m$. Exposure time: 99 ms per frame, final super-resolved frame rate: 0.9 fps.

References

(1) Lal, A.; Shan, C.; Xi, P. Structured Illumination Microscopy Image Reconstruction Algorithm. *IEEE Journal of Selected Topics in Quantum Electronics* **2016**, *22*, 50–63.

(2) Boulanger, J. SIMPLY. https://github.com/jboulanger/simply, (accessed 2025-05-28).

(3) Guizar-Sicairos, M.; Thurman, S. T.; Fienup, J. R. Efficient subpixel image registration algorithms. *Opt. Lett.* **2008**, *33*, 156–158.

(4) Manton, J. Debye diffraction code. https://github.com/jdmanton/debye_diffraction_code, (accessed 2025-05-28).
